# T122. UNMET NEEDS IN PATIENTS WITH ACUTE TRANSIENT PSYCHOTIC DISORDERS (ATPD): ANALYSIS OF PATHWAYS TO CARE: AN 8 YEARS FOLLOW-UP STUDY

**DOI:** 10.1093/schbul/sby016.398

**Published:** 2018-04-01

**Authors:** Amedeo Minichino, Grazia Rutigliano, Sergio Merlino, Cathy Davies, Dominic Oliver, Andrea De Micheli, Philip McGuire, Paolo Fusar-Poli

**Affiliations:** 1 University of Oxford; 2 Institute of Psychiatry, Psychology & Neuroscience, King’s College London; 3 University of Pisa; 4 Institute of Psychiatry Psychology and Neuroscience, King’s College London, University of Pavia; 5 Institute of Psychiatry, Psychology & Neuroscience, King’s College London, Maudsley NHS Foundation Trust, University of Pavia

## Abstract

**Background:**

Acute and transient psychotic disorders (ATPDs) constitute a highly heterogeneous category of brief psychotic disorders. The long-term course and outcomes of ATPDs is not completely clear, with more than half of patients initially diagnosed with ATPDs shifting towards other psychotic spectrum diagnoses. Uncertainties in the real-world clinical care of these patients is further complicated by the diagnostic overlap with the Brief Limited Intermittent Psychotic Symptoms (BLIPS). Thus, patients with similar diagnostic features may either be recommended conventional antipsychotic treatment (if diagnosed with ATPD and according to the current guidelines for first episode psychosis - FEP) or be contraindicated antipsychotic treatment and receive psychological therapies (recommended for BLIPS cases).

Given the complexity of the clinical presentation, admission to highly specialized services for early intervention in psychotic disorders (EIP) should represent the best therapeutic pathway for these patients. However, it is not known how many individuals with ATPDs are effectively detected and treated by EIP services.

This study aims at overcoming such a gap in knowledge by describing the pathways to care of patients with ATPDs and the treatments received across eight follow-up time-points (3, 6, 12, 18, 24, 48, 72, and 96 months).

**Methods:**

Electronic health record-based retrospective cohort study including all patients who received a first index diagnosis of ATPD (F23, ICD-10) within the South London and Maudsley (SLaM) National Health Service Trust, between 1st April 2006 and 15th June 2017. Sociodemographic and clinical characteristics were analyzed using one-way ANOVA and Tukey post-hoc tests for continuous variables and chi square test for categorical variables. Logistic regression analyses were used to investigate the association between sociodemographic characteristics and detection/treatment by EIP.

**Results:**

A total of 3074 patients receiving a first index diagnosis of ATPD (F23, ICD-10) within SLaM were included. The mean follow-up was 1495 days. After 8-year, 1883 cases (61.26%) retained the index diagnosis of ATPD; the remaining developed schizophrenia (23.8%), affective-spectrum psychoses (4.8%), and other psychotic disorders.

Only 7.5 % of ATPDs was detected and treated by Early intervention in Psychosis services (EIP). The remaining quote of patients were treated with general mental health services (91.5%).

Active treatment by EIS was more common among males, caucasian, and younger individuals (odds ratio (OR) = 1.35, 95% CI 1.01–1.7, P<0.001; OR = 0.6, 95% CI 0.46–0.78, P<0.001; and OR = 0.91, 95% CI 0.90–0.92, P<0.001, respectively).

Almost half of the total sample (48.5%) was in treatment with antipsychotic medications after 1 year of follow-up. This proportion dropped to 25% after 3 years of active treatment.

Less than 1% of ATPDs were offered psychotherapy interventions at any of the 8 time points of interest.

**Discussion:**

The present study shows that the largest majority of individuals with ATPD (91.5%) is never detected and treated by the EIP services, which should be the best clinical option for these patients.

This suggests that they are receiving neither the best first-episode care nor the best preventative care. Efforts should be done to promote outreach campaigns in general mental health services to persuade clinicians referring these patients to local EIP services, with the aim of providing the best possible care.

